# COVID‐19 Vaccine Hesitancy, Self‐Reported Adverse Events, and Determinants Among Ethiopian Healthcare Professionals During COVID‐19 Pandemic

**DOI:** 10.1002/puh2.70078

**Published:** 2025-07-25

**Authors:** Alfoalem Araba Abiye, Sisay Endale, Zenebe Negash, Shemsu Umer Hussen, Dawit Teshome Gebregeorgise, Melaku Tileku Tamiru, Dessale Abate Beyene, Hana Shafi Amde, Amanuel Yishak Negash

**Affiliations:** ^1^ Department of Pharmacology and Clinical Pharmacy School of Pharmacy College of Health Sciences Addis Ababa University Addis Ababa Ethiopia; ^2^ Department of Pharmaceutics and Social Pharmacy School of Pharmacy College of Health Sciences Addis Ababa University Addis Ababa Ethiopia; ^3^ Department of Pharmacy Asrat Woldeyes Health Science Campus Debre Berhan University Debre Berhan Ethiopia; ^4^ Department of Cardiovascular Nursing School of Nursing and Midwifery College of Health Sciences Addis Ababa University Addis Ababa Ethiopia; ^5^ Department of Anesthesiology Critical Care and Pain Medicine School of Medicine College of Health Sciences Addis Ababa University Addis Ababa Ethiopia

**Keywords:** adverse events, coronavirus disease 2019 (COVID‐19) vaccine, healthcare professionals, vaccine hesitancy

## Abstract

This nationwide cross‐sectional online survey aimed to assess coronavirus disease 2019 (COVID‐19) vaccine hesitancy, self‐reported adverse events (SRAEs), and associated determinants among Ethiopian healthcare professionals (HCPs) during the pandemic (June 19–July 31, 2021). This survey collected data from 674 HCPs after the COVID‐19 vaccine's first dose became available in Ethiopia. Hesitancy to the first and second vaccine doses was 45.6% and 17.6% among unvaccinated and vaccinated participants, respectively. Fear of side effects (39.9%) and doubts about vaccine effectiveness (29.7%) were the primary reasons for hesitancy toward the first COVID‐19 vaccine dose. For the second dose, hesitancy stemmed from perceived lack of difference between vaccinated and unvaccinated individuals (8.4%) and post‐first‐dose COVID‐19 infection (6.4%). The factors associated with hesitancy to receive the COVID‐19 vaccine (first dose) were being Muslim (*p* = 0.026), belief about the origin of COVID‐19 (*p* < 0.001), attributing the pandemic to God/Allah's wrath (*p* = 0.020), failure to maintain physical distance (*p* = 0.029), and lack of access to the first dose of the COVID‐19 vaccine (*p* < 0.001). Moreover, religion (Muslim, [*p* = 0.049]) and frequency of maintaining physical distance (i.e., often/usually, [*p* = 0.025]) were associated with second‐dose COVID‐19 vaccine hesitancy. Muslim HCPs had 63% lower odds of first‐dose hesitancy (AOR = 0.37, 95% confidence interval [CI]: 0.16–0.89). Hesitancy increased among those who never maintained physical distancing (AOR = 4.74, 95% CI: 1.18–19.08) and those with vaccine access (AOR = 4.45, 95% CI: 1.98–10.00). Pain at the injection site (55.3%) and fatigue (44.4%) were the most frequently reported SRAEs among the vaccinated HCPs. The factors associated with COVID‐19 vaccine SRAEs were religion (others) (*p* = 0.04), work experience > 7 years (*p* = 0.03), monthly income between 3000 and 10,000 (*p* = 0.03), and living with other people (*p* = 0.04). Addressing safety concerns, enhancing transparency, and leveraging religious/community engagement are critical to improving vaccine uptake among HCPs.

## Graphical abstract

Among Ethiopian HCPs, 45.6% hesitated to take the first COVID‐19 vaccine dose due to safety and efficacy concerns. Addressing these through transparent communication and religious engagement is crucial to boost vaccine uptake.

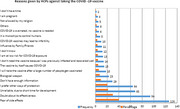

## 1 Introduction

Although the World Health Organization (WHO) no longer classifies coronavirus disease 2019 (COVID‐19) as a “global health emergency,” it remains a significant global health concern [1, 2]. Currently, vaccines such as Pfizer‐BioNTech, Moderna, AstraZeneca‐University of Oxford, Johnson and Johnson (J and J) Janssen, Russia's Sputnik, Sinovac Life Sciences, and Novavax are being used globally to mitigate the spread of the disease [3, 4]. In Ethiopia, Oxford/AstraZeneca AZD1222, Covishield, and Sinopharm BBIBP‐CorV have been approved for use [5, 6].

The SAGE (Strategic Advisory Group of Experts) Working Group on Vaccine Hesitancy defines vaccine hesitancy as “the delay in acceptance or refusal of vaccination despite the availability of vaccination services. Vaccine hesitancy is complex and context‐specific and varies across time, place, and vaccines. This was influenced by complacency, convenience, and confidence.” Complacency is a negligent behavior resulting from a lack of awareness of the potential dangers of COVID‐19 [7]. The determinants of vaccine hesitancy are complex and context‐specific and can be examined using the epidemiologic triad, considering environmental, agent, and host factors. Media and public health policies significantly contribute to vaccine refusal [8]. Perceptions of vaccine efficacy, safety, and disease susceptibility are the agent‐specific factors. The host's racial background, education level, knowledge, and experience with vaccines have been identified as factors that influence vaccine acceptance [9].

A systematic review of the COVID‐19 vaccine acceptance rate was conducted across 33 countries. The highest acceptance rates were observed in Ecuador (97.0%), Malaysia (94.3%), Indonesia (93.3%), and China (91.3%). Conversely, the lowest acceptance rates were found in Kuwait (23.6%), Jordan (28.4%), Italy (53.7%), Russia (54.9%), Poland (56.3%), the United States (56.9%), and France (58.9%). Regions such as the Middle East, North Africa, Sub‐Saharan Africa, Eastern Europe, Central Asia, and South America reported lower COVID‐19 vaccine acceptance rates among healthcare professionals (HCPs); acceptance rates varied from 27.7% in the Democratic Republic of the Congo to 78.1% in Israel [10]. A cross‐sectional study conducted at referral hospitals in the Amhara Region of Ethiopia found that 45.9% of HCPs hesitated to receive the COVID‐19 vaccine [11]. Similarly, another cross‐sectional study conducted in Addis Ababa, Ethiopia revealed that approximately 60.3% of HCPs showed vaccine hesitancy [12].

Adverse events following immunization (AEFI) were identified as contributing factors to COVID‐19 vaccine hesitancy, and their uptake was influenced by an individual's perception of vaccine safety. A recent scoping review found that individuals who received the first dose of the mRNA COVID‐19 vaccine (such as Moderna) reported mild to moderate self‐reported adverse effects (SRAEs), with a high incidence of mild effects in 65.6% of cases [13]. The most reported local AEFIs following both the first and second doses of mRNA vaccination were pain and/or paresthesia at the injection site, reported by 80.6% and 100% of participants, respectively [13]. Similarly, a study conducted in Eastern Ethiopia revealed that 84.3% of individuals experienced adverse events (AEs) after receiving the first dose of the COVISHIELD (ChAdOx1 nCoV‐19) vaccine, with injection site pain being the most prevalent symptom reported by participants (64.1%) [14]. Another study conducted on HCPs in Central Ethiopia found that 93.9% of participants experienced mild‐to‐moderate AEFIs of the COVID‐19 vaccine [15].

Acquiring knowledge about COVID‐19 vaccines and AEFIs is essential for enhancing vaccine acceptance and monitoring vaccination services. Owing to the absence of a nationally representative study, existing data on vaccine hesitancy and self‐reported AEFIs are insufficient, prompting the need for an umbrella study. Therefore, the current study aimed to systematically examine COVID‐19 vaccine hesitancy, SRAEs experiences of vaccinated individuals, and factors associated with hesitancy and AEFIs to be vaccinated in the HCP population in Ethiopia.

## 2 Methods

### 2.1 Study Area

The study was carried out in Ethiopia, the second‐most populous nation in Africa following Nigeria, with an estimated population of 123.4 million people as of 2022. Ethiopia has 11 regions and two city administrations. The health care system in Ethiopia is organized into primary, secondary, and tertiary systems. All HCPs working in city administrations and regions of Ethiopia were included in this study [16, 17].

### 2.2 Study Design and Period

A nationwide cross‐sectional online survey was conducted among Ethiopian HCPs between June 19 and July 31, 2021, following the COVID‐19 vaccine's first dose becoming available and accessible to HCPs.

### 2.3 Inclusion and Exclusion Criteria

This study included all HCPs residing in Ethiopia aged 18 years and above, regardless of whether they received the first dose of the COVID‐19 vaccine. Furthermore, participation in the online survey required internet usage and the ability to read and write in English.

### 2.4 Sample Size and Sampling Technique

This nationwide survey encompassed all eligible HCPs during the data‐collection period. The survey link was initially shared among the primary research team members through email and other social media platforms, such as Facebook, Telegram, WhatsApp, Instagram, IMO, and LinkedIn, inviting their network members to participate in the study. As a result, 684 HCPs responded within the data‐collection period. We included 674 HCPs in the final analysis by removing responses with insufficient or missing information.

### 2.5 Data Collection Tool and Procedures

A self‐administered structured questionnaire was used to collect data. Data were collected using online web‐based Google Forms [18]. The questionnaire was prepared in English, and the link was shared with study participants using social media platforms and emails. The constructed tool directed the participants to relevant questions using separate questionnaires for vaccinated and unvaccinated HCPs. The Google Forms questionnaire included a consent form and the eligibility criteria before the respondents proceeded with the study. The questionnaire was divided into two sections. Section I focused on the sociodemographic characteristics of the study participants. On the other hand, section II included questions related to comorbidities (chronic illnesses), history of COVID‐19 exposure, the practice of COVID‐19 preventive measures, sources of information about COVID‐19 disease, and, COVID‐19 vaccine, and reasons for vaccine hesitancy. Moreover, information regarding participants’ experience with the first dose of the COVID‐19 vaccine was collected, including factors influencing their decision‐making process, the time taken to decide to get vaccinated, and the frequency of SRAEs associated with the COVID‐19 vaccine. Participants who had already received the first dose of the vaccine were also asked about the specific AEs they experienced and the reasons for their hesitancy to take the second dose. AEFI were classified into mild, moderate, and severe categories on the basis of the Centers for Disease Control and Prevention (CDC) guidelines. Mild AEFI included self‐limiting symptoms requiring no medical intervention (e.g., injection site pain, mild fatigue). Moderate AEFI involved symptoms requiring medical attention but not hospitalization (e.g., fever, severe headache). Severe AEFI encompassed life‐threatening events or those requiring hospitalization (e.g., anaphylaxis or severe allergic reactions). Classification was based on participant‐reported symptoms and their reported impact.

### 2.6 Data Quality Assurance

To ensure data quality, a pretest was conducted before data collection. On the basis of the results of the pretest, appropriate corrections were made before the actual study was conducted. Throughout the data collection process, regular monitoring was conducted by the study team to ensure data completeness and consistency. To prevent multiple responses from the same participant, measures were taken using Google Forms online survey instrument. The collected data were thoroughly examined to identify incomplete entries that were subsequently excluded from the final analysis.

### 2.7 Data Analysis

Responses from Google Forms were exported to an Excel spreadsheet (2013) and were carefully reviewed for accuracy and consistency. The cleaned data were exported to SPSS version 26 for final analysis. Sociodemographic variables were analyzed using descriptive statistics such as frequencies, percentages, means, and standard deviations. After checking the assumptions, a univariate analysis was performed to obtain candidate variables for the multivariate regression model to determine the possible predictors of the dependent variable. In the bivariate analysis, factors associated with dependent variables with a *p* value of <0.2 were considered candidates for the binary logistic regression model to identify strong factors associated with dependent variables. A 95% confidence interval (CI) and a *p* value of <0.05 were used to determine statistical significance.

### 2.8 Ethical Considerations

Before participating in the study, participants were required to provide virtual written consent. The study was conducted using a Google Form, which included an informed consent section on the first page. Participants were presented with the following question at the end: “Are you willing to participate in the study?” with the response options: Yes or No.

If participants selected “No,” they were not asked any further questions and were not enrolled in the study. This ensured that only those who provided their consent continued with the survey. The informed consent was obtained virtually and was written. The text of the informed consent was included on the first page of the Google Form, allowing participants to read the details about the study before agreeing to participate. During the consent process, participants were provided with detailed information about the study's purpose, reasons for their selection, and what was expected from them. The research team assured participants that any information they provided would be treated with strict confidentiality. Privacy was maintained by avoiding the use of identifiers (such as names or phone numbers), and the data were analyzed in an aggregated form.

## 3 Results

### 3.1 Sociodemographic Characteristics of Study Participants

Of the 674 HCPs participating in this study, the majority were female (63.3%, 68.7%) and below the age of 30 years (69.4%, 51.4%) for unvaccinated and vaccinated HCPs, respectively. Among the study participants, 316 were unvaccinated and 358 were vaccinated (they received their first COVID‐19 vaccine dose). Of the unvaccinated and vaccinated HCPs, 66.3% and 51.4%, respectively, were less than 30 years old. Regarding profession, 56.3% of unvaccinated participants were pharmacists, whereas 36.6% of vaccinated participants belonged to the same profession. Regarding employment status, 54.8% of the unvaccinated participants had 6 months to 3 years of work experience, and 38.0% of the vaccinated participants had 7 or more years of work experience (Table 1).

**TABLE 1:** Sociodemographic characteristics of unvaccinated (*n* = 316) and vaccinated (*n* = 358) healthcare professionals (HCPs) in Ethiopia.

Sociodemographic variables	Unvaccinated HCPs *n* (%)	Vaccinated HCPs *n* (%)
Age (in years)		
<30	205 (64.9)	184 (51.4)
30–40	100 (31.6)	130 (36.3)
>40	11 (4.1)	44 (12.3)
Sex		
Male	116 (36.7)	112 (31.3)
Female	200 (63.3)	246 (68.7)
Religion		
Orthodox	186 (58.9)	246 (68.7)
Protestant	81 (25.6)	73 (20.4)
Muslim	41 (13.0)	27 (7.5)
Others^a^	8 (2.5)	12 (3.4)
Educational qualifications		
Bachelor's degree	176 (55.7)	145 (40.5)
Master's degree	82 (26.0)	94 (26.3)
Doctor of medicine	13 (4.1)	39 (10.9)
Diploma	21 (6.6)	31 (8.7)
Specialization degree	2 (0.6)	27 (7.5)
Others^b^	22 (7.0)	22 (6.1)
Profession		
Pharmacist	178 (56.3)	131 (36.6)
Medical doctor	19 (6.0)	71 (19.8)
Nurse	46 (14.6)	68 (19.0)
Public health officer	16 (5.1)	24 (6.7)
Medical laboratory technologist	8 (2.5)	15 (4.2)
Others^c^	49 (15.5)	49 (13.7)
Employment type		
Governmental organization	173 (54.8)	284 (79.3)
Currently not working	78 (24.7)	19 (5.3)
Non‐governmental organization	9 (2.8)	11 (3.1)
Private organization	56 (17.7)	44 (12.3)
Work experience (in years)		
<6 months	76 (24.1)	69 (19.3)
6 months–3 years	92 (29.2)	75 (20.9)
4–7 years	74 (23.5)	78 (21.8)
>7 years	73 (23.2)	136 (38.0)
Monthly income (in ETB)		
<3000	66 (22.1)	21 (5.9)
3000–10,000	178 (59.5)	200 (55.9)
10,000–30,000	49 (16.4)	125 (34.9)
>30,000	6 (2.0)	12 (3.4)
Household composition		
Alone	70 (22.2)	89 (24.9)
Family	219 (69.3)	247 (69.0)
With other people	27 (8.5)	22 (6.1)
Place of residence		
Addis Ababa	187 (59.2)	227 (63.4)
Amhara	29 (9.2)	19 (5.3)
Harari	24 (7.6)	6 (1.7)
Oromia	48 (15.2)	60 (16.8)
Sidama	3 (0.9)	11 (3.1)
Southern Nations, Nationalities, and Peoples	19 (6.0)	20 (5.6)
Others^d^	6 (1.9)	15 (4.2)

^a^ Catholic, Hawariyat, Jehovah's witness, Hindu, Wakefeta.

^b^ Doctor of dental medicine, resident, medical student (Intern), NGO: non‐government organization.

^c^ Anatomists, field epidemiologists, nutritionist, health informaticist, midwife.

^d^ Afar, Dire Dawa, Gambella, Benishangul‐Gumuz, and Somali.

### 3.2 General Health and COVID‐19‐Related Information

Regarding the general health status of the study participants, 19 (0.6%) unvaccinated and 34 (9.5%) vaccinated participants had chronic disease. The perception of health status was very good in 217 (68.7%) participants in the unvaccinated group and 259 (72.3%) in the vaccinated group. More than half, 172 (54.4%) of the unvaccinated group and 242 (67.6%) of the vaccinated group, always wore a facemask when required. In contrast, 114 (36.1%) participants in the unvaccinated group and 145 (40.5%) in the vaccinated group often used alcohol‐based hand sanitizers when necessary. Approximately 129 (40.8%) participants in the unvaccinated group and 163 (45.5%) participants in the vaccinated group maintained physical distance when necessary. More than half of the unvaccinated study participants, 163 (51.6%), believed that the COVID‐19 virus was human‐caused, but 143 (39.9%) of the study participants from the vaccinated group believed that the COVID‐19 virus had a natural origin (Table 2).

**TABLE 2:** General health and coronavirus disease 2019 (COVID‐19) related information of healthcare professionals.

General health and COVID‐19‐related variables	Unvaccinated HCPs *n* (%)	Vaccinated HCPs *n* (%)
Presence of known chronic illness		
Yes	19 (6.0)	34 (9.5)
No	297 (94)	324 (90.5)
Perception of health status		
Poor	0 (0)	3 (0.8)
Fair	10 (10)	11 (3.1)
Good	89 (28.2)	85 (23.7)
Very good	217 (68.7)	259 (72.3)
Personal or household history of COVID‐19		
Yes	89 (28.2)	96 (26.8)
No	187 (59.2)	209 (58.4)
Frequency of facemask usage when required		
Always	172 (54.4)	242 (67.6)
Often/Usually	106 (33.5)	91 (25.4)
Sometimes	38 (12.0)	23 (6.4)
Never	0 (0)	2 (0.6)
Frequency of handwashing with water and soap when required		
Always	143 (45.3)	178 (49.7)
Often/Usually	126 (39.9)	140 (39.1)
Sometimes	47 (14.9)	39 (10.9)
Never	0 (0)	1 (0.3)
Frequency of alcohol hand rub or sanitizer usage when required		
Always	91 (28.8)	133 (37.2)
Often/Usually	114 (36.1)	145 (40.5)
Sometimes	102 (32.3)	77 (21.5)
Never	9 (2.8)	3 (0.8)
Frequency of maintaining physical distance when required		
Always	38 (12.0)	56 (15.6)
Often/Usually	110 (34.8)	107 (29.9)
Sometimes	129 (40.8)	163 (45.5)
Never	39 (12.8)	32 (8.9)
Belief on the origin of COVID‐19		
Natural Source	61 (19.3)	143 (39.9)
Human‐caused	163 (51.6)	133 (37.2)
God/Allah's wrath	33 (10.4)	19 (5.3)
Others	59 (18.7)	63 (17.6)

### 3.3 Vaccine Hesitancy‐Related Information

Of the study participants, only 81 (25.6%) had access to the first dose of the COVID‐19 vaccine before the interview, and only one‐third, 102 (32.3%) of the study participants expressed willingness to receive the COVID‐19 vaccine as soon as it became available. Additionally, 172 (54.4%) participants recommended the vaccine for frontline HCPs, followed by 168 (53.2%) participants over 65 years of age (Table 3).

**TABLE 3:** Vaccine hesitancy of unvaccinated healthcare professionals in Ethiopia (*n* = 316).

Vaccine hesitancy‐related variables	*n* (%)
Access to the first dose of the COVID‐19 vaccine	
Yes	81 (25.6)
No	235 (74.4)
Willingness to receive the COVID‐19 vaccine when available	
Yes	102 (32.3)
No	144 (45.6)
Undecided	70 (22.2)
Willingness to recommend COVID‐19 vaccines to others	
Yes	201 (63.6)
No	115 (36.4)
The target population for COVID‐19 vaccine recommendations	
Frontline healthcare providers	172 (54.4)
Frontline workers (Transportation, Bank, etc.)	128 (40.5)
People above the age of 65 years	168 (53.2)
People with chronic illness/comorbidity	148 (46.8)
Children	28 (8.9)
Adult population	45 (14.2)
To all people	62 (19.6)

Abbreviation: COVID‐19, coronavirus disease 2019.

**FIGURE 1:**
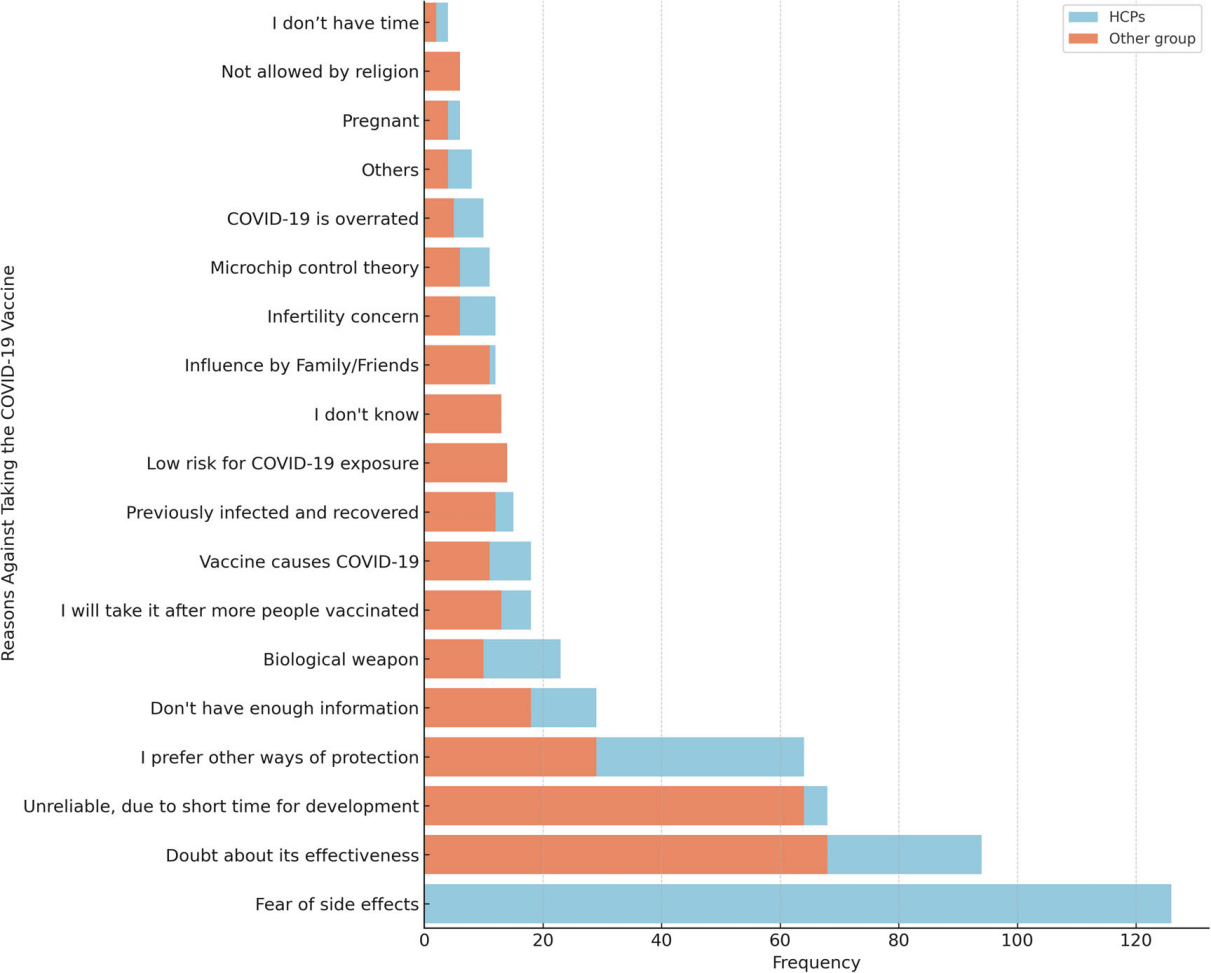
Main reasons for healthcare professionals’ non‐vaccination of COVID‐19 vaccine in Ethiopia, 2021 (*n* = 316). COVID‐19, coronavirus disease 2019.

The main reasons for vaccine refusal by HCPs were fear of AEs, 126 (39.9%), followed by doubts about the effectiveness of the vaccine, 94 (29.7%). Moreover, the vaccine was not reliable due to its short development time (68 [21.5%]), and preference for other methods of protection (64 [20.3%]) were reported reasons for COVID‐19 vaccination. Regarding information about the vaccine, 29 (9.2%) participants reported insufficient information about the COVID‐19 vaccine. Additionally, 23 (7.3%) participants believed that the vaccine was a biological weapon (Figure 1).

### 3.4 COVID‐19 Vaccination Experiences Related Information

Regarding the decision to receive the first dose of the COVID‐19 vaccine, 165 (46.1%) study participants were willing, and 295 (82.4%) expressed their willingness to receive the second dose of the COVID‐19 vaccine as soon as it became available. Moreover, more than two‐thirds, 250 (69.8%), of the study participants experienced AEFIs after the first dose of the COVID‐19 vaccine, and more than three‐fourths, 198 (79.2%) of the participants reported experiencing AEFIs within 24 h. Furthermore, 279 (77.9%) study participants wore a mask after receiving both doses (full vaccination) (Table 4).

**TABLE 4:** Coronavirus disease 2019 (COVID‐19) vaccination experiences of vaccinated healthcare professionals (HCPs) in Ethiopia (*n* = 358).

COVID‐19 vaccination experiences related variables	*N* (%) of HCPs
Decision to receive the first dose of COVID‐19 vaccine	
Very easy	165 (46.1)
Easy	110 (30.7)
Difficult	74 (20.7)
Very difficult	9 (2.5)
Time taken to decide to take the first dose of COVID‐19 vaccine availability	
Immediately	178 (49.7)
1 day	58 (16.2)
2–4 days	63 (17.6)
More than a week and above	59 (16.5)
Occurrence of AEFIs after taking the first dose of the COVID‐19 vaccine	
Yes	250 (69.8)
No	108 (30.2)
Onset of AEFI following COVID‐19 vaccine	
Within 24 h	198 (79.2)
Within 2–7 days	45 (18.0)
After a week	7 (2.8)
COVID‐19 infection after the first dose of the vaccine	
Yes	26 (7.3)
No	245 (68.4)
I don't know/I am not sure	87 (24.3)
Reasons for post‐vaccination COVID‐19 infection	
I am occupationally at risk of COVID‐19	22 (6.1)
Stopped/became reluctant to adhere to the COVID‐19 preventive measures	9 (2.5)
The first dose is not enough	48 (13.4)
The vaccine is not effective at all	12 (3.4)
Intention to receive the second dose of the COVID‐19 vaccine	
Yes	295 (82.4)
No	63 (17.6)
Reasons for not taking the second COVID‐19 vaccine dose	
I got COVID‐19 after the first dose of vaccination	23 (6.4)
I had a serious side effect after the first dose of vaccination	19 (5.3)
I took the first dose from another enforcement	9 (2.5)
I am not convinced of the difference between the vaccinated and unvaccinated individuals	30 (8.4)
Importance of mask usage after full vaccination	
Yes	279 (77.9)
No	46 (12.8)
I don't know/I am not sure	33 (9.2)
Recommendation of COVID‐19 vaccines to others	
Yes	333 (93.0)
No	25 (7.0)

Abbreviation: AEFI, adverse events following immunization.

### 3.5 Self‐Reported AEs Following COVID‐19 Vaccination

**FIGURE 2:**
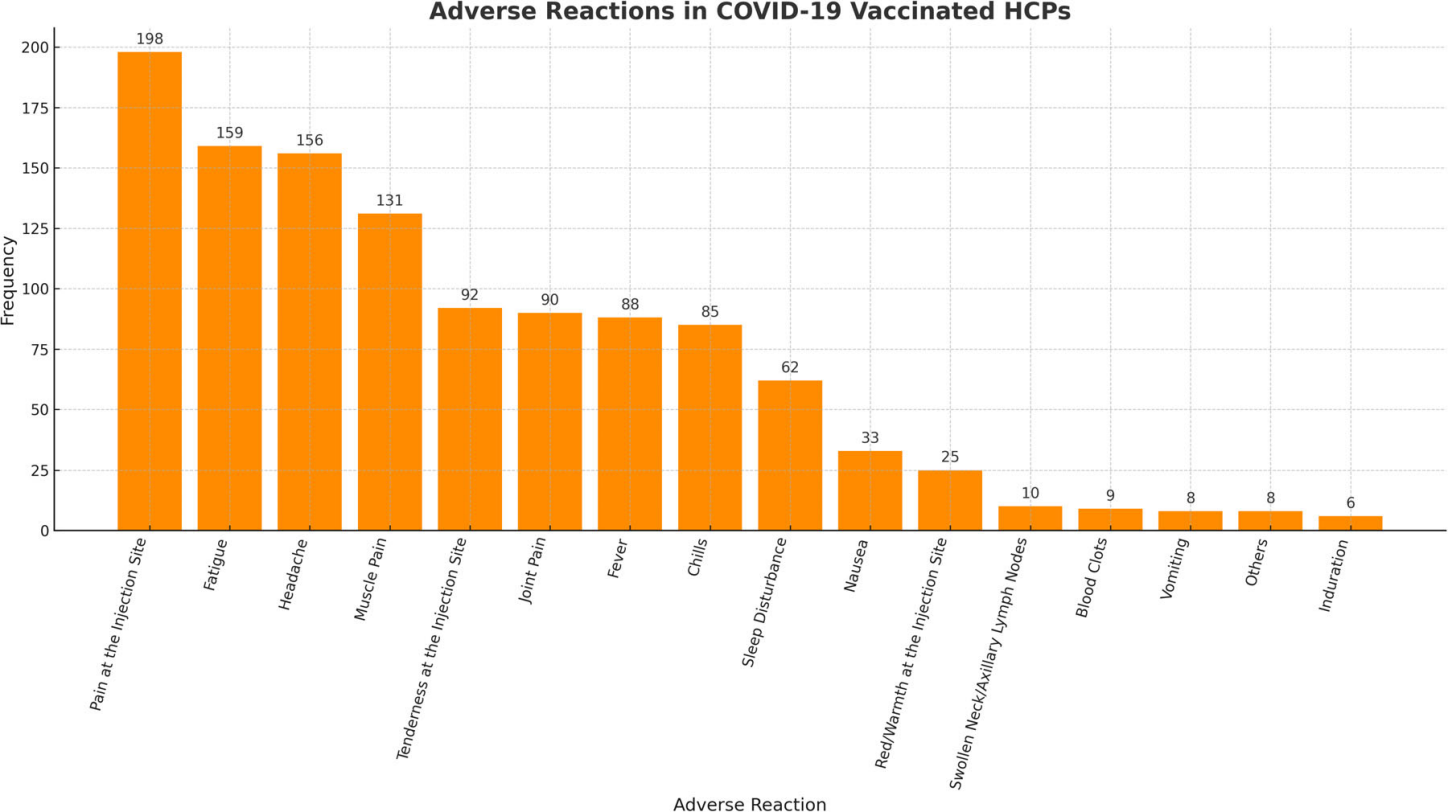
Self‐reported adverse events experienced following COVID‐19 vaccination among healthcare professionals in Ethiopia, 2021 (*n* = 316). COVID‐19, coronavirus disease 2019.

Self‐reported AEFIs were observed in 250 (69.8%) vaccinated HCPs. These were classified into mild, moderate, and severe categories per CDC guidelines. Mild AEFIs, such as injection site pain (198, 55.3%) and fatigue (159, 44.4%), were the most common. Moderate AEFI, including fever (88, 24.6%) and severe headache (156, 43.6%). Severe AEFIs, such as anaphylaxis or events requiring hospitalization, were rare. Figure 2 illustrates the frequency of AEFI by type and their distribution by severity.

### 3.6 Factors Associated With COVID‐19 Vaccine Hesitancy for the First Dose

In the univariate analysis, nine of the examined variables showed an association with COVID‐19 vaccine hesitancy. All candidate variables were categorical, with seven being multi‐categorical variables (religion, with whom one lived, perception of health status, frequency of wearing a facemask when required, maintaining physical distance when required, belief about the origin of COVID‐19 in humans, and reasons for refusing vaccination). The remaining two variables were binary (chronic diseases and access to the first dose of COVID‐19 vaccine). Nine variables were initially used for the multivariate binary regression analysis. After applying multivariate binary logistic regression methods and cross‐validating them using the hierarchical regression method, five variables were identified as associated with COVID‐19 vaccine hesitancy. The odds of COVID‐19 vaccine hesitancy were reduced by 63% among participants whose religion was Muslim (AOR = 0.37, 95% CI: 0.16–0.89, *p* = 0.026) compared with Orthodox HCPs. Additionally, the odds of vaccine hesitancy were reduced by 84% among participants who believed that the origin of COVID‐19 was man‐made (AOR = 0.16, 95% CI: 0.06–0.43, *p* < 0.0001) and by 64% among those who believed it was due to God/Allah's wrath (AOR = 0.36, 95% CI: 0.15–0.85, *p* = 0.020) compared to those who believed it had a natural source. Furthermore, the odds of vaccine hesitancy were 4.77 times higher among individuals who never maintained physical distance when required (AOR = 4.74, 95% CI: 1.18–19.08, *p* = 0.029) than among those who always maintained physical distance when required. Similarly, the odds of vaccine hesitancy were 4.45 times higher among individuals who had access to the first dose of the COVID‐19 vaccine (AOR = 4.45, 95% CI: 1.98–10.00, *p* < 0.0001) compared to those who had no access to the first dose (Table 5).

**TABLE 5:** Factors associated with coronavirus disease 2019 (COVID‐19) vaccine hesitancy among healthcare professionals (HCPs) in Ethiopia (*n* = 316).

		Vaccine hesitancy				
Variables	Category	Yes (*n*)	No (*n*)	COR of 95% CI	AOR of 95% CI	*p* value
Religion	Orthodox	55	131	1	1	
	Protestant	25	56	0.94 (0.53–1.66)	0.88 (0.44–1.76)	0.721
	Muslim	18	23	0.54 (0.27–1.07)	0.37 (0.16–0.89)	0.026*
	Others^a^	4	4	0.42 (0.10–1.74)	0.56 (0.08–3.94)	0.562
Chronic illness	No	91	206	1	1	
	Yes	11	8	0.32 (0.13–0.83)	0.16 (0.05–0.56)	0.004*
Maintain physical distance when required	Always	15	23	1	1	0.114
	Often/usually	39	71	1.19 (0.56–2.54)	1.22 (0.48–3.11)	0.672
	Sometimes	43	86	1.30 (0.62–2.75)	1.20 (0.46–3.14)	0.705
	Never	5	34	4.44 (1.42–13.89)	4.74 (1.18–19.08)	0.029*
Beliefs about the origin of COVID‐19	Natural source	31	30	1	1	
	Man‐made	54	109	2.09 (1.15–3.79)	0.16 (0.06–0.43)	0.0001*
	God/Allah's wrath	6	27	4.65 (1.68–12.86)	0.36 (0.15–0.85)	0.020*
	Others^b^	11	48	4.51 (1.98–10.29)	1.02 (0.26–3.95)	0.981
Access to the first dose of the COVID‐19 vaccine	No	91	144	1	1	
	Yes	11	70	4.02 (2.02–8.00)	4.45 (1.98–10.00)	0.0001*

Abbreviations: AOR, adjusted odds ratio, COR, crude odds ratio.

^a^ Others are Catholic, Hawariyat, Jehovah's Witness, Hindu, Wakefeta.

^b^ Others = I don't know, animals, Chinese virus, no comment.

^*^ Statistical significance at *p* < 0.05.

### 3.7 Factors Associated With COVID‐19 Vaccine AEFI

In the univariate analysis, seven of the examined variables showed an association with AEFIs related to the COVID‐19 vaccine. Among these variables, religion, total years of work experience, monthly income, and residence were identified as being associated with AEs related to the COVID‐19 vaccine. The odds of reporting COVID‐19 vaccine AEFIs were reduced by 90% in participants belonging to a different religion compared with orthodox HCPs (AOR = 0.09, 95% CI: 0.01–0.95, *p* = 0.04). In addition, HCPs with more than 7 years of experience (AOR = 2.19, 95% CI: 1.09–4.38, *p* = 0.03) were 2.19 times more likely to report COVID‐19 vaccine AEFIs than HCPs with less than 6 months of experience. In addition, COVID‐19 vaccine AEFIs were 77% less likely to be reported among individuals who lived with others (AOR = 0.23, 95% CI: 0.06–0.91, *p* = 0.04) than among individuals who lived alone. Similarly, individuals with monthly incomes between 3000 and 10,000 ETB (AOR = 0.12, 95% CI: 0.02–0.82, *p* = 0.03) were 88% less likely to report COVID‐19 vaccine AEFIs than those with monthly incomes less than 3000 ETB (Table 6).

**TABLE 6:** Factors associated with coronavirus disease 2019 (COVID‐19) vaccine self‐reported adverse effects (SRAEs) among healthcare professional (HCPs) (*n* = 358).

		Vaccine AEFIs				
Variables	Category	Yes	No	COR of 95% CI	AOR of 95% CI	*p* value
Religion	Orthodox	167	79	1	1	
	Protestant	56	17	0.19 (0.02–1.52)	0.14 (0.02–1.19)	0.07
	Muslim	16	11	0.30 (0.04–2.50)	0.19 (0.02–1.72)	0.14
	Others^a^	11	1	0.13 (0.02–1.18)	0.09 (0.01–0.95)	0.04*
Total years of work experience	<6 months	47	22	1	1	
	6 months–3 years	54	21	1.20 (0.65–2.23)	1.08 (0.52–2.27)	0.83
	4–7 years	62	16	1.45 (0.78–2.68)	1.24 (0.61–2.53)	0.55
	>7 years	87	49	2.18 (1.14–4.19)	2.19 (1.09–4.38)	0.03*
Monthly income in ETB	<3000	10	11	1	1	
	3000–10,000	146	54	0.18 (0.03–1.04)	0.12 (0.02–0.82)	0.03*
	10,000–30,000	84	41	0.54 (0.12–2.55)	0.67 (0.13–3.34)	0.62
	>30,000	10	2	0.41 (0.09–1.96)	0.56 (0.11–2.92)	0.49
With whom do you live	Alone	65	24	1	1	0.11
	Family	166	81	0.43 (0.12–1.57)	0.27 (0.07–1.11)	0.07
	With other people	19	3	0.32 (0.09–1.13)	0.23 (0.06–0.91)	0.04*

Abbreviations: AEFI, adverse events following immunization; AOR, adjusted odds ratio; CI, confidence interval; COR, crude odds ratio.

^a^Others Catholic, Hawariyat, Jehovah's Witness, Hindu, and Wakefeta.

^*^ Statistical significance at *p* < 0.05.

### 3.8 Factors Associated to Receive the Second Dose of the COVID‐19 Vaccine

In univariate analysis, variables such as religion, educational status, perception of health status, frequency of maintaining physical distance when required, and chronic diseases were evaluated. Among these, only religion and frequency of maintaining physical distance were significantly associated with receiving the second‐dose COVID‐19 vaccine. The odds of hesitancy to the second dose of COVID‐19 were 88% lower in participants with a Muslim religion (AOR = 0.12, 95% CI: 0.01–0.99, *p* = 0.049) than in orthodox HCPs. On the other hand, the odds of hesitating to take the second dose of COVID‐19 of the vaccine were 4.23 times higher in persons of other religions (AOR = 4.23, 95% CI: 1.00–17.86, *p* = 0.049). In addition, the odds of hesitancy to the second dose of the COVID‐19 vaccine were 69% lower among persons who often or usually kept physical distance when needed (AOR = 0.31, 95% CI: 0.11–0.86, *p* = 0.025) than among persons who always kept physical distance when needed (Table 7).

**TABLE 7:** Factors associated to receive a second dose of the coronavirus disease 2019 (COVID‐19) vaccine among healthcare professionals (HCPs) (*n* = 358).

		Vaccine AEFIs				
Variables	Categories	Yes	No	COR of 95% CI	AOR of 95% CI	*p* value
Religion	Orthodox	199	47	1	1	0.048*
	Protestant	62	11	0.47 (0.14–1.65)	0.90 (0.41–2.01)	0.803
	Muslim	26	1	0.36 (0.09–1.38)	0.12 (0.01–0.99)	0.049*
	Others^a^	8	4	0.08 (0.01–0.79)	4.23 (1.00–17.86)	0.049*
Educational status (highest level completed)	Bachelor's Degree	114	31	1	1	0.14
	Master's Degree	72	22	2.72 (0.60–12.27)	1.29 (0.65–2.58)	0.45
	Doctor of Medicine	35	4	3.06 (0.66–14.11)	0.48 (0.13–1.78)	0.27
	Diploma	28	3	1.14 (0.19–6.81)	0.34 (0.09–1.33)	0.12
	Specialization Degree	26	1	1.07 (0.16–7.01)	0.26 (0.03–2.08)	0.20
	Others^b^	20	2	0.38 (0.03–4.55)	0.34 (0.07–1.78)	0.20
Presence of known chronic illness	No	264	60	1	1	
	Yes	31	3	2.35 (0.69–7.94)	0.38 (0.10–1.40)	0.15
Perception of health status	Fair	9	2	1	1	0.37
	Good	71	14	1.06 (0.22–5.06)	1.30 (0.22–7.59)	0.77
	Poor	1	2	0.93 (0.49–1.81)	17.85 (0.58–547.84)	0.09
	Very good	214	45	9.51 (0.84–107.16)	1.29 (0.23–7.11)	0.77
Frequency of maintaining physical distance when required	Always			1	1	0.063
	Never			1.15 (0.55–2.37)	0.52 (0.14–1.95)	0.330
	Often/Usually			0.70 (0.25–1.96)	0.31 (0.11–0.86)	0.025*
	Sometime			0.44 (0.21–0.90)	0.85 (0.36–2.03)	0.72

Abbreviations: AEFI, adverse events following immunization; AOR, adjusted odds ratio; CI, confidence interval; COR, crude odds ratio.

^a^ Catholic, Hawariyat, Jehovah's Witness, Hindu, Wakefeta.

^b^ Doctor of Dental Medicine, Resident, and Medical Student (Intern).

^*^ Statistical significance at *p* < 0.05.

## 4 Discussion

This national survey aimed to examine the prevalence of COVID‐19 vaccine hesitancy, SRAE experiences of vaccinated individuals, and factors associated with hesitancy and AEFIs among Ethiopian HCPs. The existing literature indicates that concerns regarding vaccine safety and efficacy are among the factors contributing to vaccine hesitancy among the public [19]. Notably, hesitancy among HCPs poses a significant barrier to the successful implementation of vaccination programs [20]. Addressing this issue is crucial, because increasing hesitancy can lead to a decline in vaccination coverage and potentially contribute to infectious disease outbreaks. Given their unique position, HCPs have a vested interest in receiving vaccinations that positively affect the overall health of the population.

The findings of this national survey showed that 214 (67.6%) HCPs were hesitant to receive the first dose of the COVID‐19 vaccine, and the hesitancy in this survey was higher than that in studies conducted in the Amhara region of northwestern Ethiopia (45.9%) [11], Dessie (58.29%) [21], and Addis Ababa (60.3%) [22]. This may be because the survey was conducted in the early phase of the vaccination program, when the efficacy and safety data of the vaccines were still being collected at the time of the survey, resulting in HCPs being uncertain about the safety and efficacy of the vaccine owing to its short development time. Additionally, 60.1% of HCPs in Sierra Leone [23], 50.5% in Nigeria [24], and 41.0% in South Africa [25] were reluctant to use the COVID‐19 vaccine.

Among the vaccinated HCPs, 63 (17.6%) of the study participants were unwilling to receive the second dose of the COVID‐19 vaccine, and this proportion was higher than that in the study conducted in Togo (10.9%)[26]. This could be due to the fear of side effects, as in this survey, a large proportion of the respondents, 250 (69.8%) experienced at least one side effect with the first dose of the COVID‐19 vaccine. Another assumption could be that 26 (7.3%) HCPs in this survey were infected with COVID‐19 after receiving their first dose of the COVID‐19 vaccine.

The main reason cited by HCPs for hesitating to be vaccinated was fear of side effects, 126 (39.9%), and in other studies conducted in Dessie (53.23%) [21], Qatar (28%) [27], and the United States of America (60%) [28], they expressed strong fears of unpredictable side effects of COVID‐19 vaccines. This might be due to the loss of confidence in the reliability of the vaccine because of the short duration of the study and the lack of comprehensive short‐ and long‐term safety data. This was evidenced by 68 (21.5%) HCPs in this survey, who considered the COVID‐19 vaccine unreliable in terms of its short‐ and long‐term safety owing to the short development time of the vaccine. In this survey, 94 (29.7%) HCPs had doubts about the effectiveness of the COVID‐19 vaccine, as evidenced by 64 (20.3%) preferring other methods of protection against COVID‐19 vaccination. In another study conducted in Western Ethiopia, 173 (40.14%) HCPs disagreed with the efficacy of the COVID‐19 vaccine, and 63 (14.62%) disagreed [29]. In a study conducted in Addis Ababa, 275 (45.2%) agreed that acquiring natural immunity to COVID‐19 (by contracting the disease) was better than vaccination [22]. Another qualitative study conducted in Addis Ababa also suggested that HCPs were reluctant to be vaccinated against COVID‐19 because of the undefined duration of vaccine protection and risk of reinfection [30].

A total of 250 (69.8%) participants in this survey reported at least one adverse reaction after the first dose of the COVID‐19 vaccine, which was higher than that reported by Dessie (56.98%) [21]. However, it was lower than that COVID‐19 AEFIs reported in the Czech Republic (93.1%) [31], Togo (71.6%) [26], Western Ethiopia (84.3%) [14], the United Kingdom systemic AEFIs (13.5%), and local AEFIs (71.9%) [32]. In this survey, the most frequently reported AEFIs were injection site pain 198 (55.3%), fatigue: 159 (44.4%), headache: 156 (43.6%), and muscle pain: 131 (36.6%]). In the Czech Republic, the most common AEFIs was that 93.1% of participants reported at least one AEFI after vaccination with COVID‐19 [31], and in Togo, the most commonly reported AEFIs were injection site pain (91.0%), asthenia (74.3%), headache (68.7%), soreness (55.0%), and fever (47.5%) [26]. In addition, in western Ethiopia, injection site pain (64.1%), fatigue (35.7%), headache (28.9%), joint pain (26.5%), and muscle pain (21.5%) [14], and in Dessie Hospital, fever (44.44%), headache (39.03%), fatigue (27.35%), injection site pain (25.93%), and nausea (24.22%) [21] were the frequently reported adverse effect. In a study conducted in Iraq Pain at the injection site (74%), muscle pain (37%), headache (31.2%), and fever (26.8%) were reported as the most common AEFIs for COVID‐19 vaccines [33]. In contrast, less than 30% of HCPs complained of injection site pain, and less than 25% complained of fatigue and headache after the first dose of COVID‐19 vaccines, but 57.2% reported tenderness at the injection site in the United Kingdom [32].

Classifying AEFI by severity per CDC guidelines revealed that the majority of reported events were mild (e.g., injection site pain and fatigue), consistent with global studies on COVID‐19 vaccines [26, 31]. Moderate AEFIs, such as fever or severe headache, were less frequent, and severe AEFIs, such as anaphylaxis, were rare, aligning with the safety profile of vaccines like AstraZeneca and Sinopharm used in Ethiopia. This stratification enhances the understanding of vaccine safety among HCPs, highlighting that most AEs are transient and manageable. However, the fear of even mild AEFI contributed to hesitancy, underscoring the need for targeted education to address safety concerns.

HCPs play a critical role in the control and prevention of COVID‐19 by providing frontline care to patients, informing preventive measures, and serving as role models in the community. In this survey, religion was found to be associated with hesitation regarding vaccination. This may be because, in this study, 33 (10.4%) HCPs believed that the origin of the COVID‐19 virus was God/Allah. Therefore, many HCPs were reluctant to be vaccinated and may have been concerned about the incompatibility of the vaccine with religious beliefs [34]. It has been observed that people who do not maintain physical distance and those who have debated the sources of COVID‐19 are more likely to be hesitant to be vaccinated. This could be due to negative attitudes towards COVID‐19 and preventive measures. However, HCPs with chronic diseases were 84% less likely to be hesitant to receive the COVID‐19 vaccine. The results of this study are consistent with those reported in Italy [35] and Egypt [36]. This may be because patients with chronic diseases have a greater risk of severe sequelae or death due to COVID‐19 and are therefore more likely to be vaccinated, as shown by studies demonstrating that multiple comorbidities increase the risk of death from COVID‐19 [37].

The HCPs who had access to the first dose COVID‐19 vaccine were 4.45 times more hesitant (AOR = 4.45, 95% CI: 1.98–10.00, *p* < 0.0001) than those who did not have access to the first dose of COVID‐19 vaccine. In contrast, male sex, older age, advanced education level, higher knowledge level, history of influenza vaccination compliance, and perceived risk and severity of COVID‐19 have been reported in many studies as facilitating factors for the uptake of COVID‐19 vaccination [38, 39, 40, 41, 42, 43, 44, 45]. However, in this survey, the independent predictors of unwillingness to receive a second dose of the COVID‐19 vaccine were religion and maintaining physical distance when required. Another study conducted in the Oromia region of Ethiopia found that moderate physical activity and fear of vaccination were independent factors related to reported AEFIs [46]. In addition, HCPs with more than 7 years of experience (AOR = 2.19, 95% CI: 1.09–4.38, *p* = 0.03) were 2.19 times more likely to report COVID‐19 vaccine AEFIs than HCPs with less than 6 months of experience. Since this survey was conducted 3–4 months after Ethiopia's vaccine rollout (March 2021), a period marked by limited safety data and public uncertainty, potentially contributing to heightened hesitancy compared to later studies.

Given the continued threat of COVID‐19, mitigating the health effects of the pandemic requires high levels of vaccine acceptance by HCPs, as they are front‐liners (often in high‐risk encounters) and provide face‐to‐face services. These requirements are met through the use of safe and effective vaccines. Knowledge and experience of adverse reactions to COVID‐19 vaccines are critical factors for accepting and delivering vaccines and advocates for patient immunization. Therefore, continued provision of reliable scientific evidence about COVID‐19 vaccines, underlying risks and benefits, and awareness‐generating activities through varied communication strategies is essential for addressing hesitancy, concerns, and fears and increasing trust and acceptance. Moreover, COVID‐19 vaccine hesitancy and adverse effects may spill over to increase hesitancy in routine vaccination programs. The integration of COVID‐19 vaccination into the existing health systems is crucial for effective vaccine delivery. The ongoing COVID‐19 pandemic further supports the need for joint safety monitoring, reporting mechanisms for COVID‐19, and routine vaccination.

Finally, the national survey has policy implications. First, our survey results indicated the importance of accurate and transparent COVID‐19 information. This aligns with CDC's emphasis on finding credible vaccine information; HCPs must be exposed to credible and accurate COVID‐19 information to make informed decisions. Second, our survey results may help to inform revised policies within healthcare facilities. Improving working conditions, implementing adequate COVID‐19 strategies, and strengthening employer‐employee communication could potentially benefit vaccine uptake in the long run.

### 4.1 Limitation of the Study

Although the study did not explicitly assess religious campaigns, Orthodox and Muslim leaders in Ethiopia publicly endorsed vaccination during the study period. This may explain the protective association among Muslim HCPs. We acknowledge this as a limitation and recommend future research on religious influences.

## 5 Conclusion

COVID‐19 vaccine hesitancy among Ethiopian HCPs was driven by safety concerns and efficacy doubts. Protective factors included Muslim faith and chronic illness, whereas hesitancy correlated with distrust in origin narratives and lax distancing. Targeted education, transparent communication, and interfaith collaboration are vital to improving vaccine confidence.

## Author Contributions

All authors made a significant contribution to the work reported, whether in the conception, study design, execution, acquisition of data, analysis, and interpretation, or in all these areas, took part in drafting, revising, or critically reviewing the article and gave final approval of the version to be published.

## Ethics Statement

Ethical approval was obtained from the Ethical Review Board of the School of Pharmacy, College of Health Sciences, Addis Ababa University (ERB/SOP/290/13/2021).

## Consent

Informed consent was obtained from all subjects involved in the study.

## Conflicts of Interest

The authors declare no conflicts of interest.

## Data Availability

The data that support the findings of this study are available from the corresponding author upon reasonable request.
